# Enhanced safety surveillance of GSK's quadrivalent seasonal influenza vaccine in Germany and Spain (2021/2022 season) using an electronic patient‐reported outcome system for vaccine safety remote monitoring

**DOI:** 10.1111/irv.13098

**Published:** 2023-03-26

**Authors:** Gaël Dos Santos, Tamara Eckermann, Xavier Martínez‐Gómez, Jose Parra, Ugo Nwoji, Ignacio Salamanca de la Cueva

**Affiliations:** ^1^ GSK Wavre Belgium; ^2^ Hausarztpraxis Heimeranplatz Munich Germany; ^3^ Hospital Universitari Vall d'Hebron Barcelona Spain; ^4^ GSK Rockville Maryland USA; ^5^ Instituto Hispalense de Pediatría Sevilla Spain

**Keywords:** influenza, post‐marketing surveillance, safety, vaccination

## Abstract

**Background:**

Seasonal influenza epidemics are managed through vaccination each winter in the European Union, to prevent infections, complications, and deaths. As circulating virus strains vary unpredictably, vaccines are reformulated annually, and their safety monitored rapidly and continuously at the start of each season, following European Medicines Agency guidelines.Seasonal influenza epidemics are managed through vaccination each winter in the European Union, to prevent infections, complications, and deaths. As circulating virus strains vary unpredictably, vaccines are reformulated annually, and their safety monitored rapidly and continuously at the start of each season, following European Medicines Agency guidelines.

**Methods:**

This enhanced safety surveillance study assessed pre‐specified and other adverse events (AEs) occurring within 7 days of GSK's inactivated quadrivalent seasonal influenza vaccine (IIV4) in children and adults in Spain and Germany. As the study was conducted during the COVID‐19 pandemic (2021/2022 season), data were collected electronically, using a web portal or call center.

**Results:**

Safety was assessed in 737 participants (median age 49 and 9 years in Germany and Spain, respectively, 19.3% with a chronic medical condition). After Dose 1 and Dose 2, respectively, 332 (45.1%) and 5 (26.3%) participants reported at least one AE, primarily pre‐specified AEs. The most common AEs after Dose 1 (adults and children) were injection site pain, swelling or erythema, headache, and fatigue. After Dose 2 (in children), the most common AEs were injection site pain, rhinorrhea, fatigue, and decreased appetite. No new or unexpected safety issues were identified.

**Conclusion:**

This study supports and confirms the safety profile of GSK's IIV4 in all age groups with a vaccine indication. The new electronic safety reporting method (with response rates of 75.4% following Dose 1 and 100% following Dose 2) provides an alternative for future studies to reduce the burden on sites or in case site visits are not feasible.

## 1 INTRODUCTION

In Europe, seasonal influenza epidemics occur each winter, typically between November and April, and affect all age groups. Uncomplicated cases can experience fever, headache, muscle pain, sore throat, and cough, lasting days to weeks. Severe cases or secondary bacterial infections can lead to pneumonia, encephalitis, or myocarditis and can be fatal, especially in vulnerable populations, for example, chronic conditions, older adults, and infants.^1^

Influenza remains a major public health burden^1^, ^2^, ^3^; however, annual vaccination plays a pivotal role in reducing the risk of infection and complications.^4^ Influenza types A and B viruses, and their respective A subtypes and B lineages, cause most disease, and circulating strains vary unpredictably each winter.^5^ Due to frequent genetic and antigenic changes in influenza viruses,^6^ seasonal vaccines are reformulated with updated viral strains and therefore need constant benefit–risk monitoring.

To comply with European Medicines Agency requirements,^7^, ^8^ following close dialog with regulators of the European Union,^9^ GSK launched a series of enhanced safety surveillance (ESS) studies from the 2015/2016 season onwards.^10^, ^11^, ^12^, ^13^ The present ESS study of GSK's inactivated quadrivalent seasonal influenza vaccine (IIV4) was implemented in Germany and Spain to monitor the frequency and severity of adverse events (AEs) experienced within 7 days following vaccination in near real time. This year, in anticipation of potential COVID‐19 challenges to the study, data were collected using an electronic Case Report Form (e‐CRF) completed by investigators and participants via an electronic Patient Reported Outcome (e‐PRO) web‐based portal or with help from a call center.

**FIGURE 1:**
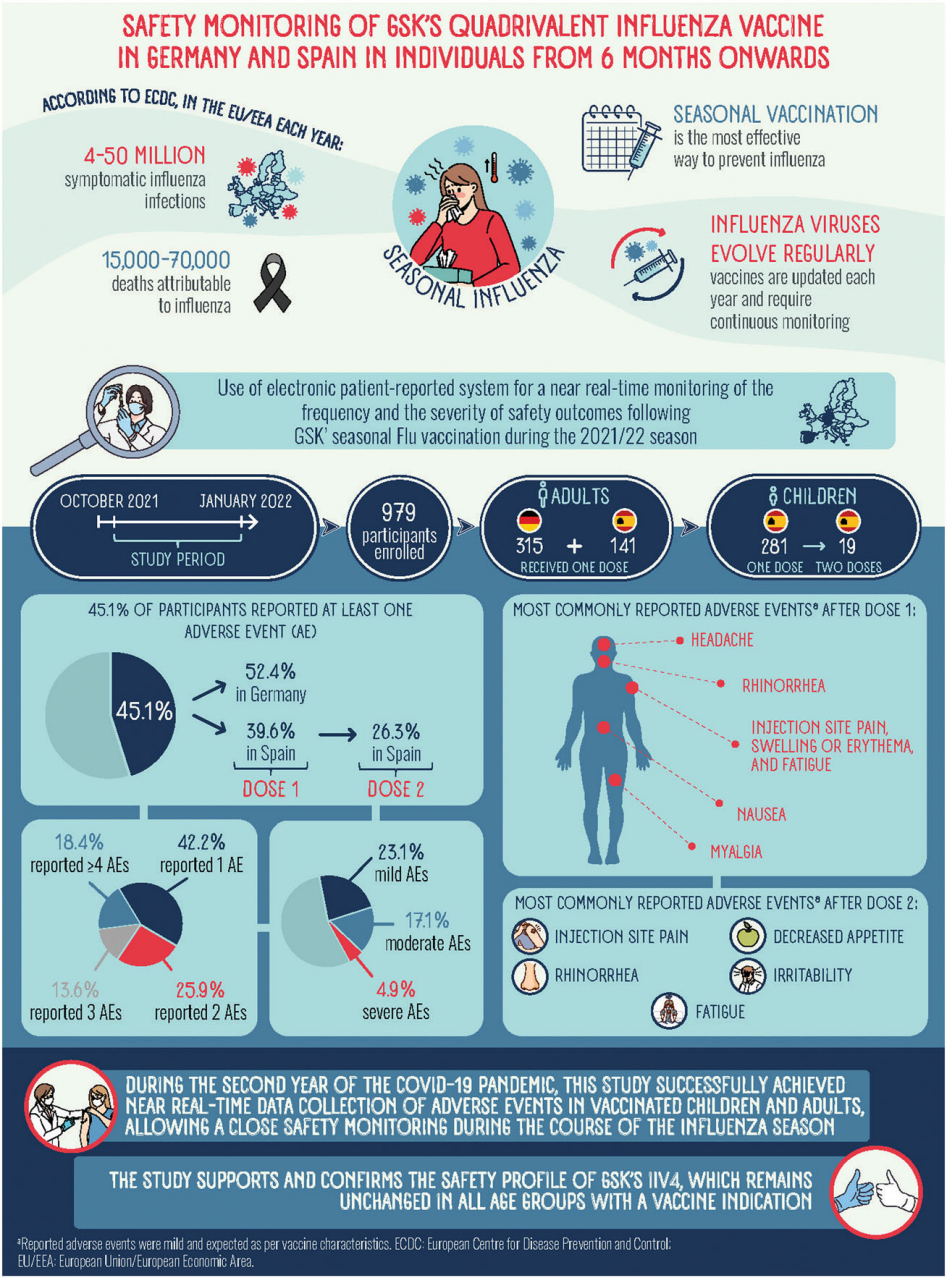
Graphical plain language summary.

The primary objective was to estimate the cumulative percentage of participants reporting pre‐specified adverse events of interest (AEIs) and any other AEs occurring within 7 days of vaccination with GSK's IIV4 in each country and overall. As this study was conducted during the COVID‐19 pandemic, potential logistical, site access, and medical staff constraints were anticipated. This study was thus also intended to provide information about potential effects of the pandemic on the reporting of influenza vaccine safety. Figure 1 presents a graphical plain language summary.

## 2 METHODS

### 2.1 Study design and population

This prospective ESS study of GSK's IIV4 (*Influsplit Tetra* in Germany, *Fluarix Tetra* in Spain ‐ GSK study ID 213828) was conducted between October 1, 2021 and January 7, 2022. ESS studies aim to identify potential safety signals early on, and the use of electronic reporting in this study may help to inform future safety surveillance studies for influenza in Europe. Pre‐specified AEIs were considered as well as open fields to report any non‐listed AEs or the absence of any AEs.

Participants, or legal representatives, provided written informed consent prior to enrolment and were vaccinated with GSK's IIV4 according to routine country‐specific practices and local guidelines.^14^ A second dose of GSK's IIV4 was administered only to children aged <9 years who have not previously been vaccinated against influenza. Participants completed the adverse drug reaction (ADR) questionnaire (for type and severity of AEs) and a COVID‐19 assessment form (for history of COVID‐19 infections as well as associated signs and symptoms) using a web‐based e‐PRO portal or with help from a call center. Healthcare professionals (HCPs) were to report any serious AE (SAE) that they deemed related to GSK's IIV4 within 24 h of becoming aware of it, using the electronic reporting system.

The goal was to enroll approximately 1000 participants from five HCPs across Germany and Spain (from October 1 up to December 31, 2021) and to include participants in all age groups for which IIV4 is indicated, that is, from 6 months of age. Recruitment targeted participants aged over 6 months in Spain and adults aged 18 years and older in Germany. Children in care were not eligible for this study.

### 2.2 Statistical methods

All analyses were descriptive. The analysis of AEs was performed on the Safety Set, which included all enrolled vaccinated participants who initiated activities to receive the login details for the web portal, or contact details for the call center, to complete the ADR.

Demographic characteristics and risk status for influenza‐associated morbidity and mortality were summarized using frequency tables (*n*, %) for categorical variables and mean, standard deviation, median, minimum, and maximum for continuous data.

The number and percentage of participants who received co‐administered vaccination on the same day as the GSK's IIV4 were tabulated by vaccination class. The percentage of participants who completed the ADR card and COVID‐19 form was tabulated by center/country.

For each vaccine dose, the cumulative percentage of participants reporting AEs within 7 days of vaccination was estimated each week from study start (i.e., using International Standards Organization [ISO] weeks 39 to 52) and using the Medical Dictionary for Regulatory Activities (MedDRA^15^), Primary System Organ Class (SOC), and Preferred Term (PT). In addition, for each vaccine dose, the weekly and cumulative percentages of participants reporting AEs were estimated by age strata (6 months to 17 years; 18 to 65 years; >65 years) and risk status (at risk/not at risk) for each country. These secondary objectives are not presented here but will be available in the GSK clinical trial register (GSK Study ID 213828)^16^ by March 2023.

The cumulative percentages of participants reporting AEs by severity grade were also calculated over the whole study period.

The 95% confidence intervals (CIs) accounting for the clustering effect of centers were computed on all estimated percentages (extended Clopper–Pearson exact CI for clustered data). The design effects and the intracluster correlation coefficients were also estimated for the cumulative percentage of participants reporting AEs by MedDRA Primary SOC and PT within 7 days post‐each vaccination using the completed ADR questionnaire, over the whole study period.

### 2.3 Ethical considerations

Ethical approval preceded participant enrolment. The following were consulted in line with country requirements and approved the study protocol: Bayerische Landesärztekammer: Central Ethics Committee in Germany (approval received on June 28, 2021), Comité Coordinador de Ética de la Investigación Biomédica de Andalucía: Regional Regulatory Authority in Spain (approval received on June 18, 2021), and CEIC Autonómico de Andalucía: Central Ethics Committee in Spain (approval received on June 18, 2021).

## 3 RESULTS

### 3.1 Participant disposition and characteristics

**FIGURE 2:**
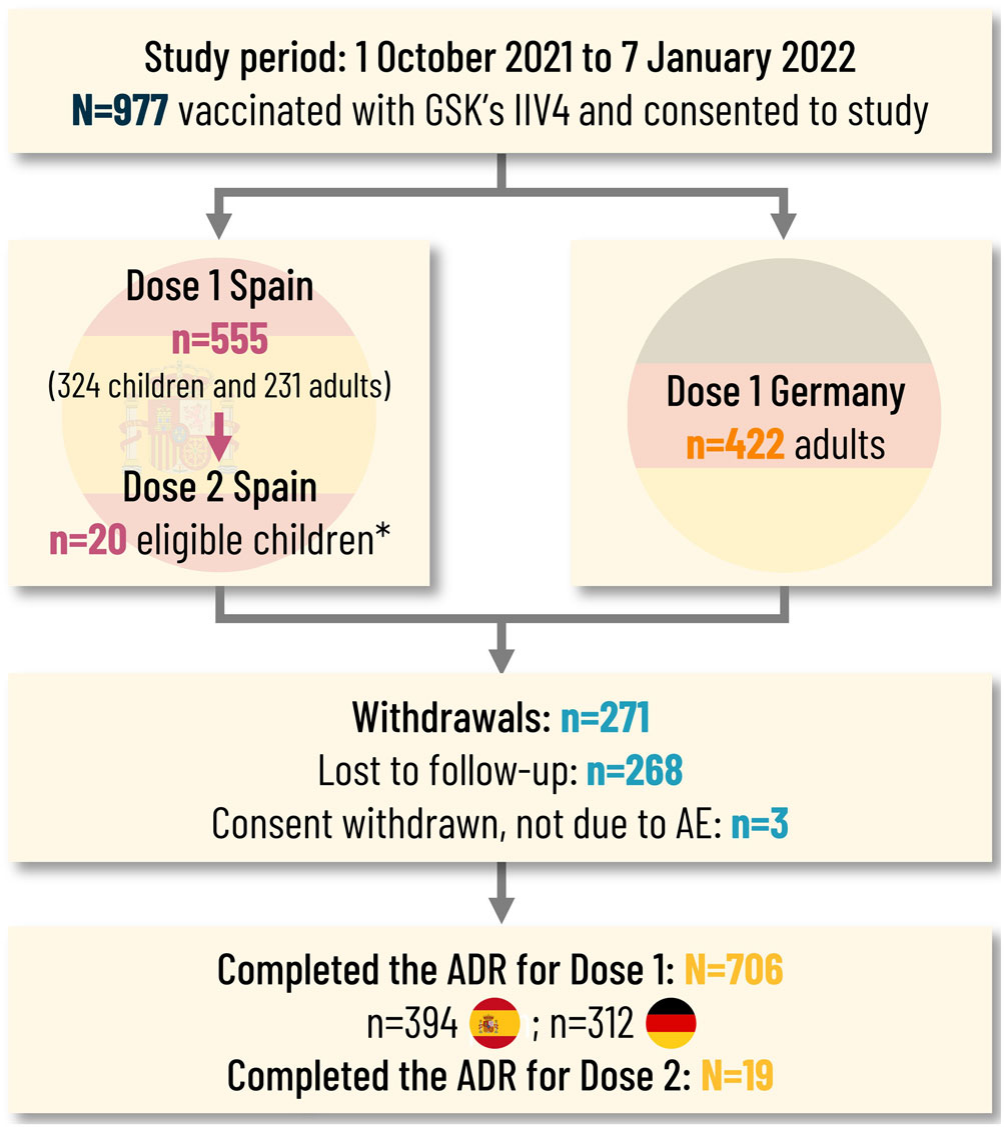
Attrition diagram. *Children eligible for Dose 2 were ≤9 years of age at inclusion and receiving seasonal influenza vaccine for the first time. ADR, adverse drug reaction (card); AE, adverse event; IIV4, inactivated quadrivalent seasonal influenza vaccine; N, n, total number, number in subcategory.

Over the study period (October 1, 2021 and January 7, 2022), 979 vaccinees receiving GSK's IIV4 were enrolled in the study, of which two had an invalid informed consent form. Of the remaining 977 enrolled participants who consented to the study, there were 422 adults in Germany and 555 participants in Spain (324 children [<18 years] and 231 adults). Among the children, 20 were eligible for a second dose. Overall, 268 participants were lost to follow‐up, and three withdrew consent but not due to an AE (Figure 2). In total, 706 (72.3%) participants completed the study (i.e., completed the ADR). The Safety Set included 737 participants (315 in Germany and 422 in Spain) for the analyses, including 36 participants who did not complete the ADR or did not document the presence or absence of AEs.

The median age of participants was 49 years in Germany and 9 years in Spain, with overall 38.1%, 56.9%, and 5.0% in the age categories <18 years, 18–65 years, and >65 years, respectively (Table 1, Safety Set). Most participants (96.2%) were Caucasian, and 57.0% of participants overall were not considered at risk for influenza‐associated morbidity and mortality based on healthcare provider's judgment. The most common risk factors^1^, ^17^ of being infected or developing severe complications encompass a chronic medical condition (in 19.3%) and being a healthcare worker (in 15.2%) (Table 1). The most common vaccine classes co‐administered with IIV4 were other viral vaccines (i.e., COVID‐19 vaccines) in Germany (in 10.9% of participants) and pneumococcal (in 5.6%) and hepatitis vaccines (in 4.5%) in Spain (Table S1).

**TABLE 1:** Demographic characteristics and risk status (Dose 1, Safety Set).

	Germany *N* = 315		Spain *N* = 422		Total *N* = 737	
**Age at Dose 1**						
Mean (SD), years	48.82 (14.25)		18.67 (19.61)		31.55 (23.01)	
Median (range), years	49.0 (18.0–85.0)		9.0 (0.6–64.0)		35.0 (0.6–85.0)	

	*n*	%	*n*	%	*n*	%
**Age category at Dose 1**						
6 months to 17 years	0	0	281	66.6	281	38.1
18 to 65 years	278	88.3	141	33.4	419	56.9
>65 years	37	11.7	0	0	37	5.0
**Gender**						
Female	180	57.1	218	51.7	398	54.0
Male	135	42.9	204	48.3	339	46.0
**Geographic ancestry**						
White ‐ Caucasian/European	310	98.4	399	94.5	709	96.2
White ‐ Arabic/North African	2	0.6	8	1.9	10	1.4
Black or African American	0	0	2	0.5	2	0.3
American Indian or Alaska Native	0	0	2	0.5	2	0.3
Asian ‐ Southeast Asian	2	0.6	4	0.9	6	0.8
Asian ‐ Central/South Asian	1	0.3	1	0.2	2	0.3
Asian ‐ East Asian	0	0	1	0.2	1	0.1
Asian ‐ Japanese	0	0	0	0	0	0
Native Hawaiian, other Pacific Islander	0	0	0	0	0	0
Other	0	0	5	1.2	5	0.7
**Risk status for influenza‐associated morbidity and mortality** ^a^						
At risk	94	29.8	223	52.8	317	43.0
Not at risk	221	70.2	199	47.2	420	57.0
**Risk group for influenza‐associated morbidity and mortality** ^a^ ^,^ ^b^						
Older adult (≥65 years old)	40	12.7	0	0	40	5.4
Age ≥6 months with chronic medical conditions	50	15.9	92	21.8	142	19.3
Condition affecting respiratory function	3	1.0	16	3.8	19	2.6
Immunosuppression from disease or treatment	0	0	37	8.8	37	5.0
Resident in long‐term care home	0	0	0	0	0	0
Healthcare worker	35	11.1	77	18.2	112	15.2
Pregnant woman	0	0	1	0.2	1	0.1
Child 6–23 months old	0	0	26	6.2	26	3.5
Other	0	0	1	0.2	1	0.1

Abbreviations: HCP, healthcare professional; *N*, *n*, total number, number in subcategory; SD, standard deviation.

^a^ Assessed by the HCP based on medical judgment and experience.

^b^ Participants may be assigned to more than one risk group; Five participants reported “Other” Geographic ancestry in Spain (Other = “Hispanic or Latino”).

### 3.2 AEs

**FIGURE 3:**
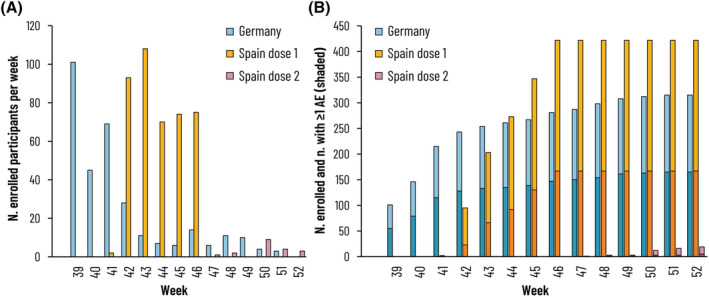
(A) Enrolled participants (number) by week, dose, and country. (B) Cumulative enrolled participants with proportion reporting ≥1 AE (shaded) after Dose 1 or Dose 2 (Safety Set). AE, adverse event; N, n, total number, number in subgroup. Figure 3A shows the number of participants enrolled in each country, by dose, each week. Participants in Germany were adults and therefore received one dose of GSK's inactivated quadrivalent influenza vaccine, while some participants in Spain were children eligible for two doses. Figure 3B shows the cumulative number of enrolled participants by week and country, showing in darker shades the number reporting at least one AE within 7 days of Dose 1 or Dose 2.

Participants were enrolled in ISO weeks 39 to 52 (i.e., between October 1, 2021 and December 31, 2021) (Figure 3A), and 332 participants (45.1%) reported an AE, with 52.4% in Germany and 39.6% in Spain reporting at least one AE after Dose 1 and 26.3% reporting at least one AE after Dose 2 (Figure 3B).

After Dose 1 (administered to 737 participants), 332 participants (45.1%) reported an AE, mostly from the pre‐specified AEIs. Among these, 42.2% reported one AE, while 25.9%, 13.6%, and 18.4% reported two, three, or four or more AEs, respectively. The percentage of participants who experienced mild, moderate, and severe AEs was 23.1%, 17.1%, and 4.9%, respectively.

The most frequently reported MedDRA SOCs were general and administration site disorders reported by 39.8% of participants (e.g., mainly injection site pain, swelling or erythema, and fatigue); nervous system disorders by 11.3% (e.g., mostly headache); respiratory, thoracic, and mediastinal disorders by 6.1% (e.g., mostly rhinorrhea); gastrointestinal disorders by 6.0% (e.g., nausea); and musculoskeletal and connective tissue disorders in 5.7% (e.g., myalgia) (Table 2). AEs by MedDRA SOC reported by fewer than 5% of participants included psychiatric disorders (i.e., irritability), skin and subcutaneous tissue disorders (e.g., hyperhidrosis), metabolism and nutrition disorders (i.e., decreased appetite), immune system disorders (e.g., nasopharyngitis), blood and lymphatic system disorders (e.g., lymphadenopathy), and eye disorders and ear and labyrinth disorders (Table 2).

**TABLE 2:** Cumulative participants (%) reporting AEIs (in green) and/or other AEs post‐Dose 1 over study period (Safety Set).

MedDRA primary system organ class (code) Preferred term (code)	Germany *N* = 315	Spain *N* = 422	Total *N* = 737
	*n* (%)95% CI, LL–UL	*n* (%)95% CI, LL–UL	*n* (%)95% CI, LL–UL
**Any**	**165 (52.4)** **32.8–71.5**	**167 (39.6)** **0.1–99.3**	**332 (45.1)** **29.0–61.9**
**Any general disorders and administration site conditions (10018065)**	**147 (46.7)** **25.1–69.2**	**146 (34.6)** **0.0–99.2**	**293 (39.8)** **24.2–57**
Injection site pain (10022086)	120 (38.1) 10.5–73.1	114 (27.0) 0.0–97.5	234 (31.8) 16.8–50.1
Fatigue (10016256)	57 (18.1) 6.5–36.5	27 (6.4) 4.3–9.2	84 (11.4) 3.7–24.8
Injection site swelling (10053425)	32 (10.2) 7.1–14.0	32 (7.6) 5.2–10.5	64 (8.7) 6.8–11.0
Injection site erythema (10022061)	22 (7.0) 4.4–10.4	15 (3.6) 0.0–66.6	37 (5.0) 2.4–9.0
Pyrexia (10037660)	7 (2.2) 0.1–10.1	21 (5.0) 3.1–7.5	28 (3.8) 1.6–7.5
Chills (10008531)	13 (4.1) 0.4–14.8	3 (0.7) 0.0–85.4	16 (2.2) 0.2–8.0
Swelling face (10042682)	0 (0.0) 0.0–1.2	0 (0.0) 0.0–0.9	0 (0.0) 0.0–0.5
Influenza like illness (10022004)	1 (0.3) 0.0–7.7	0 (0.0) 0.0–0.9	1 (0.1) 0.0–1.5
**Any nervous system disorders (10029205)**	**51 (16.2)** **8.2–27.5**	**32 (7.6)** **5.2–10.5**	**83 (11.3)** **5.5–19.8**
Headache (10019211)	42 (13.3) 3.0–33.6	30 (7.1) 4.9–10.0	72 (9.8) 5.1–16.6
Somnolence (10041349)	12 (3.8) 2.0–6.6	2 (0.5) 0.1–1.7	14 (1.9) 0.3–6.3
Dizziness (10013573)	8 (2.5) 1.1–4.9	2 (0.5) 0.1–1.7	10 (1.4) 0.2–4.4
Burning sensation (10006784), sciatica (10039674)	*n* = 1; 1NR	*n* = 0; 0NR	1 (0.1) eachNR
**Any respiratory, thoracic, and mediastinal disorders (10038738)**	**18 (5.7)** **3.4–8.9**	**27 (6.4)** **0.0–70.8**	**45 (6.1)** **3.5–9.8**
Rhinorrhea (10039101)	15 (4.8) 2.7–7.7	27 (6.4) 0.0–70.8	42 (5.7) 2.9–9.8
Cough (10011224)	1 (0.3) 0.0–1.8	4 (1.0) 0.3–2.4	5 (0.7) 0.2–1.6
Oropharyngeal pain (10068319), increased upper airway secretion (10062717)	3; 0NR	0; 1NR	3 (0.4); 1 (0.1) NR
**Any gastrointestinal disorders (10017947)**	**20 (6.4)** **1.5–16.6**	**24 (5.7)** **0.0–73.3**	**44 (6.0)** **3.4–9.7**
Nausea (10028813)	12 (3.8) 0.1–21.2	10 (2.4) 1.1–4.3	22 (3.0) 1.2–6
Abdominal pain (10000081)	3 (1.0) 0.2–2.8	11 (2.6) 0.0–67.4	14 (1.9) 0.3–5.8
Diarrhea (10012735)	3 (1.0) 0.2–2.8	8 (1.90) 0.8–3.7	11 (1.5) 0.8–2.7
Vomiting (10047700)	0 (0.0) 0.0–1.2	3 (0.7) 0.2–2.1	3 (0.4) 0.1–1.2
Lip swelling (10024570), swollen tongue (10042727)	1; 1NR	0; 0NR	1 (0.1) eachNR
Dyschezia (10051244), dyspepsia (10013946)	1; 1NR	0; 0NR	1 (0.1) eachNR
**Any musculoskeletal and connective tissue disorders (10028395)**	**31 (9.8)** **6.8–13.7**	**11 (2.6)** **0.0–77.4**	**42 (5.7)** **1.2–16.0**
Myalgia (10028411)	23 (7.3) 4.7–10.8	8 (1.9) 0–96.8	31 (4.2) 0.6–13.7
Arthralgia (10003239)	14 (4.4) 2.5–7.3	5 (1.2) 0.4–2.7	19 (2.6) 0.7–6.3
Neck pain (10028836), pain in extremity (10033425)	1; 1NR	0; 0NR	1 (0.1) eachNR
**Any psychiatric disorders (10037175)**	**3 (1.0)** **0.0–8.3**	**12 (2.8)** **0.0–89.2**	**15 (2.0)** **0.2–8.0**
Irritability (10022998)	3 (1.0) 0.0–8.3	12 (2.8) 0.0–89.2	15 (2.0) 0.2–8.0
**Any skin and subcutaneous tissue disorders (10040785)**	**12 (3.8)** **0.2–17.5**	**2 (0.5)** **0.1–1.7**	**14 (1.9)** **0.2–7.0**
Hyperhidrosis (10020642)	11 (3.5) 0.1–16.6	1 (0.2) 0.0–1.3	12 (1.6) 0.1–7.0
Pruritus (10037087), rash (10037844)	1; 0NR	0; 1NR	1 (0.1) eachNR
Erythema (10015150)	1 (0.3) 0.0–10.2	0 (0.0) 0.0–0.9	1 (0.1) 0.0–1.7
**Any metabolism and nutrition disorders (10027433)**	**5 (1.6)** **0.5–3.7**	**8 (1.9)** **0.0–45.9**	**13 (1.8)** **0.9–3.0**
Decreased appetite (10061428)	5 (1.6) 0.5–3.7	8 (1.9) 0.0–45.9	13 (1.8) 0.9–3.0
**Any infections and infestations (10021881)**	**4 (1.3)** **0.0–10.9**	**1 (0.2)** **0.0–1.3**	**5 (0.7)** **0.0–3.7**
Nasopharyngitis (10028810), tonsillitis (10044008), bronchitis (10006451)	3; 1; 0NR	0; 0; 1NR	3 (0.4); 1 (0.1) both NR
**Any blood and lymphatic system disorders (10005329)**	**2 (0.6)** **0.1–2.3**	**2 (0.5)** **0.1–1.7**	**4 (0.5)** **0.2–1.4**
Lymphadenopathy (10025197), lymph node pain (10025182)	2; 0NR	1; 1NR	3 (0.4); 1 (0.1) NR
**Any immune system disorders (10021428)**	**0 (0.0)** **0.0–1.2**	**1 (0.2)** **0.0–1.3**	**1 (0.1)** **0.0–0.8**
Hypersensitivity (10020751)	0 (0.0) 0.0–1.2	1 (0.2) 0.0–1.3	1 (0.1) 0.0–0.8
**Any eye disorders (10015919)**	**1 (0.3)** **0.0–7.7**	**0 (0.0)** **0.0–0.9**	**1 (0.1)** **0.0–1.5**
Eye allergy (10015907)	1 (0.3) 0.0–7.7	0 (0.0) 0.0–0.9	1 (0.1) 0.0–1.5
**Any ear and labyrinth disorders (10013993)**	**1 (0.3)** **0.0–1.8**	**0 (0.0)** **0.0–0.9**	**1 (0.1)** **0.0–0.8**
Ear pain (10014020)	1 (0.3) 0.0–1.8	0 (0.0) 0.0–0.9	1 (0.1) 0.0–0.8

*Note*: In green are pre‐specified AEIs. AEs that occurred in three or fewer cases were grouped to simplify the table, with the number of cases presented for each symptom and the total number (%) for each symptom; the individual 95% CI were not reported (NR).

Abbreviations: 95% CI, 95% confidence interval (extended Clopper–Pearson exact CI for cluster data); AE, adverse event; AEI, adverse event of interest; LL, lower limit; MedDRA, Medical Dictionary for Regulatory Activities; *N*, total number of participants; *n* (%), number (percentage) of participants reporting the symptom at least once; UL, upper limit.

The most frequently reported AEs, by at least 5% of participants, following Dose 1 were (by MedDRA PT) as follows: injection site pain (31.8%), fatigue (11.4%), headache (9.8%), injection site swelling (8.7%), and injection site erythema (5%) (Table 2).

After Dose 2 (administered in 19 children), five participants (26.3%) reported AEIs: two participants experienced one AE, while one participant each experienced two, three, and four AEs, respectively. Two participants each had mild and moderate AEs, respectively, and one had severe AEs. Overall, after Dose 1 and Dose 2 in participants who had two doses, nine (47.4%) reported no AEs, while four, two, and four participants, respectively, reported one, three, and four or more AEs.

General disorders and administration site conditions were the most commonly reported MedDRA SOCs (i.e., three participants with injection site pain and two with fatigue), followed by respiratory, thoracic, and mediastinal disorders (i.e., three participants with rhinorrhea); metabolism and nutrition disorders (i.e., two participants with decreased appetite); and psychiatric disorders (i.e., one participant with irritability) (Table 3).

**TABLE 3:** Cumulative participants (%) reporting AEIs (in green) and/or other AEs post‐Dose 2 over study period (Safety Set).

MedDRA primary system organ class (code) Preferred term (code)	Spain *N* = 19	
	*n*	% [95% CI, LL–UL]
**Any**	**5**	**26.3 [9.2–51.2]**
**Any general disorders and administration site conditions (10018065)**	**4**	**21.1 [6.1–45.6]**
Injection site pain (10022086)	3	15.8 [3.4–39.6]
Fatigue (10016256)	2	10.5 [1.3–33.1]
Pyrexia (10037660), Chills (10008531), Swelling face (10042682), Injection site erythema (10022061) or swelling (10053425)	0	0.0 [0.0–17.7]
**Any respiratory, thoracic, and mediastinal disorders (10038738)**	**3**	**15.8 [3.4–39.6]**
Rhinorrhoea (10039101)	3	15.8 [3.4–39.6]
**Any metabolism and nutrition disorders (10027433)**	**2**	**10.5 [1.3–33.1]**
Decreased appetite (10061428)	2	10.5 [1.3–33.1]
**Any psychiatric disorders (10037175)**	**1**	**5.3 [0.1–26.0]**
Irritability (10022998)	1	5.3 [0.1–26.0]
**Any gastrointestinal disorders (10017947)**	**0**	**0.0 [0.0–17.7]**
Abdominal pain (10000081), diarrhea (10012735), lip swelling (10024570), nausea (10028813), swollen tongue (10042727), vomiting (10047700)	0	0.0 [0.0–17.7]
**Any eye disorders (10015919)**	**0**	**0.0 [0.0–17.7]**
Eye allergy (10015907)	0	0.0 [0.0–17.7]
**Any musculoskeletal and connective tissue disorders (10028395)**	**0**	**0.0 [0.0–17.7]**
Arthralgia (10003239), Myalgia (10028411)	0	0.0 [0.0–17.7]
**Any skin and subcutaneous tissue disorders (10040785)**	**0**	**0.0 [0.0–17.7]**
Hyperhidrosis (10020642), pruritus (10037087), rash (10037844)	0	0.0 [0.0–17.7]
**Any immune system disorders (10021428)**	**0**	**0.0 [0.0–17.7]**
Hypersensitivity (10020751)	0	0.0 [0.0–17.7]
**Any nervous system disorders (10029205)**	**0**	**0.0 [0.0–17.7]**
Dizziness (10013573), headache (10019211) or somnolence (10041349)	0	0.0 [0.0–17.7]

*Note*: In green are pre‐specified AEIs.

Abbreviations: 95% CI, 95% confidence interval (extended Clopper‐Pearson exact CI for cluster data); AE, adverse event; AEI, adverse event of interest; LL, lower limit; MedDRA, Medical Dictionary for Regulatory Activities; *N*, total number of participants; *n* (%), number (percentage) of participants reporting the symptom at least once; UL, upper limit.

The most frequently reported AEs following Dose 2 were (by MedDRA PT): injection site pain (15.8%), followed by rhinorrhea (15.8%), fatigue (10.5%), decreased appetite (10.5%), and irritability (5.3%) (Table 3).

Following any vaccine dose in Spain (administered in 422 participants), 170 participants (40.3%) reported AEs. The percentage of participants who experienced mild, moderate, and severe AEs, respectively, was 20.9% (88/422), 13.7% (58/422), and 5.7% (24/422).

Overall, 35.6% of participants reported general and administration site conditions; 7.6% nervous system disorders (e.g., mostly headache); 6.6% respiratory, thoracic, and mediastinal disorders (e.g., mostly rhinorrhea); and 5.7% gastrointestinal disorders (mainly abdominal pain and nausea). Other AEs (e.g., irritability, myalgia, and decreased appetite) were less frequently reported (Table 4).

**TABLE 4:** Cumulative participants (%) reporting AEIs (in green) and/or other AEs post‐doses 1 and 2 over study period—Spain (Safety Set).

MedDRA primary system organ class (code) Preferred term (code)	Spain *N* = 422		
	AE, *n* ^a^	*n*	% [95% CI, LL–UL]
**Any**	**360**	**170**	**40.3 [0.2–98.7]**
**Any general disorders and administration site conditions (10018065)**	**217**	**150**	**35.6 [0.1–98.3]**
Injection site pain (10022086)	117	117	27.7 [0.0–95.7]
Injection site swelling (10053425)	32	32	7.6 [5.2–10.5]
Fatigue (10016256)	29	29	6.9 [4.7–9.7]
Pyrexia (10037660)	21	21	5.0 [3.1–7.5]
Injection site erythema (10022061)	15	15	3.6 [0.0–66.6]
Chills (10008531)	3	3	0.7 [0.0–85.4]
Swelling face (10042682)	0	0	0.0 [0.0–0.9]
**Any nervous system disorders (10029205)**	**34**	**32**	**7.6 [5.2–10.5]**
Headache (10019211)	30	30	7.1 [4.9–10.0]
Dizziness (10013573)	2	2	0.5 [0.1–1.7]
Somnolence (10041349)	2	2	0.5 [0.1–1.7]
**Any respiratory, thoracic, and mediastinal disorders (10038738)**	**35**	**28**	**6.6 [0.0–74.8]**
Rhinorrhea (10039101)	30	28	6.6 [0.0–74.8]
Cough (10011224)	4	4	1.0 [0.3–2.4]
Increased upper airway secretion (10062717)	1	1	0.2 [0.0–47.0]
**Any gastrointestinal disorders (10017947)**	**32**	**24**	**5.7 [0.0–73.3]**
Abdominal pain (10000081)	11	11	2.6 [0.0–67.4]
Nausea (10028813)	10	10	2.4 [1.1–4.3]
Diarrhea (10012735)	8	8	1.9 [0.8–3.7]
Vomiting (10047700)	3	3	0.7 [0.2–2.1]
Lip swelling (10024570) or swollen tongue (10042727)	0	0	0.0 [0.0–0.9]
**Any psychiatric disorders (10037175)**	**13**	**12**	**2.8 [0.0–89.2]**
Irritability (10022998)	13	12	2.8 [0.0–89.2]
**Any musculoskeletal and connective tissue disorders (10028395)**	**13**	**11**	**2.6 [0.0–77.4]**
Myalgia (10028411)	8	8	1.9 [0.0–96.8]
Arthralgia (10003239)	5	5	1.2 [0.4–2.7]
**Any metabolism and nutrition disorders (10027433)**	**10**	**10**	**2.4 [0.0–61.2]**
Decreased appetite (10061428)	10	10	2.4 [0.0–61.2]
**Any skin and subcutaneous tissue disorders (10040785)**	**2**	**2**	**0.5 [0.1–1.7]**
Rash (10037844)	1	1	0.2 [0.0–47.0]
Hyperhidrosis (10020642)	1	1	0.2 [0.0–1.3]
Pruritus (10037087)	0	0	0.0 [0.0–0.9]
**Any blood and lymphatic system disorders (10005329)**	**2**	**2**	**0.5 [0.1–1.7]**
Lymph node pain (10025182)	1	1	0.2 [0.0–47.0]
Lymphadenopathy (10025197)	1	1	0.2 [0.0–1.3]
**Any immune system disorders (10021428)**	**1**	**1**	**0.2 [0.0–1.3]**
Hypersensitivity (10020751)	1	1	0.2 [0.0–1.3]
**Any infections and infestations (10021881)**	**1**	**1**	**0.2 [0.0–1.3]**
Bronchitis (10006451)	1	1	0.2 [0.0–1.3]
**Any eye disorders (10015919)**	**0**	**0**	**0.0 [0.0–0.9]**
Eye allergy (10015907)	0	0	0.0 [0.0–0.9]

*Note*: In green are pre‐specified AEIs.

Abbreviations: 95% CI = 95% confidence interval (extended Clopper‐Pearson exact CI for cluster data); AE, adverse event; AEI, adverse event of interest; LL, lower limit; MedDRA, Medical Dictionary for Regulatory Activities; *N*, total number of participants; *n* ^a^, number of specified AEs reported; *n* (%), number (percentage) of participants reporting the AE at least once; UL, upper limit.

No deaths occurred, and no serious AEs were reported that were deemed by the HCP to be related to GSK's IIV4. No participants reported any of the following pre‐specified AEIs: febrile convulsion, anaphylactic reaction, Bell's palsy, or Guillain–Barre syndrome after any dose. There was one report of hypersensitivity after Dose 1 in a child.

### 3.3 Analysis of COVID‐19 impact on the study

Before the study, from January 1, 2021, there were 23 confirmed cases of COVID‐19 (2.4%) reported (7 in Germany and 16 in Spain) and 1 probable (0.10%) and 2 suspected (0.20%) cases in Spain. During the study, there were two confirmed cases (0.40%), one probable case (0.20%), and one suspected case (0.2%) of COVID‐19 in Spain. Among these four participants, two participants had cough, one had sore throat, one had nausea, and one was asymptomatic. From January 1, 2021 to the end of the study, 64 participants (6.60%) received at least one dose of a COVID‐19 vaccine (48 in Germany and 16 in Spain). Among the participants with history of COVID‐19 vaccination; 51.7% (15/29) in Germany and 36.4% (4/11) in Spain reported AEs after Dose 1, while none reported AEs after Dose 2.

## 4 DISCUSSION

This study rapidly collected and monitored safety data from participants vaccinated with GSK's IIV4, from individuals aged over 6 months. The study provides an overview of types of AEs experienced and frequency by country and age group.

As with all previous ESS studies starting with the 2014/2015 season,^10^, ^11^, ^12^, ^13^ no safety concerns arose during or at the end of this study. No new or unexpected AEs were reported, and all reported AEs were within the scope of events and the expected event rates for the populations studied, based on clinical data in GSK's IIV4 Summary of Product Characteristics (SmPC); that is, incidence rates (frequency) of AEs were of similar or lower magnitude than those in the SmPC.^18^

The electronic ADR (using the e‐PRO web‐based platform or call center) was completed by 75.4% of participants following Dose 1 and 100% following Dose 2, which was lower than previous IIV4 ESS studies using paper cards.^13^, ^19^ Some participants/site staff found the electronic platform difficult to use, despite supportive documentation and training. Per protocol, the call center option could not be used by minors, even with parental supervision, which may have posed logistical issues for some sites/participants. Although the electronic ADR completion rate was lower than traditional active studies,^20^ or web‐based intensive monitoring,^21^ the completion rate in this study falls within the range observed in other surveillance studies using an e‐PRO system.^22^, ^23^, ^24^ Therefore, e‐PRO systems can still be used to collect safety information, for example, to limit the burden of work for sites or to monitor safety when a site visit is not possible, for example, during COVID‐19 lockdowns.

Systematic collection of medical history associated with COVID‐19 infections and symptoms allowed us to better appreciate and empirically observe any potential interference with the vaccination process and IIV4 safety profile. Ultimately, findings were aligned with previous seasons and did not differ from the last season which also occurred during the COVID‐19 pandemic. Co‐administration of any other vaccines on the same day as GSK's IIV4 was also captured; however, due to the relatively limited numbers reported (i.e., 11.2% for Dose 1 and none for Dose 2), it was challenging to observe or evaluate trends or impact on frequency of AEs experienced.

The study bears some limitations. As Belgian sites declined to participate this year, recruitment targets in Germany and Spain were increased to reach the required sample size. Spanish sites were able to enroll participants on a two‐dose vaccination schedule (i.e., children <9 years old not previously vaccinated against influenza), to cover all ages indicated for GSK's IIV4. Limitations in the e‐PRO system and associated e‐CRF meant it was not possible to directly identify participants who had received the login details or call center contact details as planned. Therefore, as a proxy at the analysis stage, these participants were identified if they initiated the first activities to complete the ADR questionnaire (i.e., either opening the web‐based ADR questionnaire or contacting the call center). The e‐PRO system did not allow data related to AEs entered in the system to be queried (e.g., date typos). Despite these limitations, ESS studies contribute to the continuous monitoring of vaccine safety and complement routine pharmacovigilance, as discussed extensively elsewhere.^9^

The study also encompasses several strengths. The study captured all age groups with an indication for vaccination with GSK's IIV4, including the pediatric population, with a fairly balanced distribution between age groups. The inclusion of participants from different countries and age groups allowed different vaccination practices and diversity in population characteristics to be captured. The electronic application used to collect standardized data facilitated data extraction, cleaning, and analysis with the objective of maximizing near real‐time assessment. Therefore, the timely electronic data encoding by the medical team allowed for continuous assessment, for example, weekly review of AEs, and prompt investigation if necessary. Furthermore, ongoing review of events allowed prompt clarification from centers via individual queries.

## 5 CONCLUSIONS

This study for GSK's IIV4 was conducted in the 2021/2022 influenza season allowing near real‐time safety monitoring at the start of the influenza season. Among the children, adults, and older adult participants included, there were no safety signals detected that could affect public health. This study supports and confirms the safety profile of GSK's IIV4, which remains unchanged in all age groups with a vaccine indication. A new approach using web‐based or call center assisted electronic safety reporting was implemented, with potential future uses as a compromise approach if site visit restrictions apply (e.g., during lockdowns).

## AUTHOR CONTRIBUTIONS

**Gaël Dos Santos:** Conceptualization; formal analysis; funding acquisition; methodology; project administration; supervision; validation; visualization; writing‐original draft; writing‐review and editing. **Tamara Eckermann:** Conceptualization; formal analysis; project administration; resources; supervision; writing‐review and editing. **Xavier Martínez‑Gómez:** Conceptualization; formal analysis; project administration; resources; supervision; writing‐review and editing. **Jose Parra:** Conceptualization; data curation; formal analysis; methodology; software; validation; writing‐review and editing. **Ugo Nwoji:** Conceptualization; methodology; writing‐review and editing. **Ignacio Salamanca de la Cueva:** Conceptualization; formal analysis; project administration; resources; supervision; writing‐review and editing.

## CONFLICT OF INTEREST STATEMENT

Gaël Dos Santos is employed by GSK and holds shares in GSK. Tamara Eckermann declares that her institution received payment from GSK for the conduct of the study. Jose Parra and Ugo Nwoji were employed by GSK at the time of the study. Xavier Martínez‐Gómez declares having received payment from GSK for slide materials and from AstraZeneca for lecture, outside of the submitted work. Ignacio Salamanca de la Cueva declares that his institution received payment from GSK for the conduct of the study, by contract approved by the corresponding ethical committees and health authorities, and outside of this study, for other trials from other vaccine manufacturers. He has also received grants/honoraria as a consultant/advisor/speaker for attending conferences and practical courses from GSK and other vaccine manufacturers. The authors declare no other financial and non‐financial relationships and activities.

## ETHICS STATEMENT

All procedures performed in studies involving human participants were in accordance with the ethical standards of the institutional and/or national research committees and with the 1964 Helsinki declaration and its later amendments or comparable ethical standards.

The following IECs or IRBs and Regional Authorities were consulted in line with country requirements: Commissie Medische Ethiek UZ/KU Leuven (Ethics Committee [EC]) in Belgium (approval received on August 26, 2020), Ethik‐Kommission der Bayerischen (EC) in Germany (approval received on September 1, 2020), and in Spain the Comité Coordinador de Ética de la Investigación Biomédica de Andalucía (EC) and autonomous community for Andalusia (approval received on July 23, 2020) and for Catalonia (approval received on September 25, 2020). In the respective countries, the ethical approval preceded the study participants' enrolment, with the first participant enrolled on October 1, 2020.

## PATIENT CONSENT STATEMENT

Informed consent was obtained from all individual participants included in the study.

## TRADEMARKS

*Influsplit Tetra* and *Fluarix Tetra* are trademarks owned by or licensed to GSK.

## Supporting information


**Table S1.** Vaccines co‐administered with GSK's IIV4

## Data Availability

Anonymized individual participant data and study documents can be requested for further research (www.clinicalstudydatarequest.com).
