# Supplementary material for: Enhanced safety surveillance of GSK's quadrivalent seasonal influenza vaccine in Germany and Spain (2021/2022 season) using an electronic patient‐reported outcome system for vaccine safety remote monitoring
Source: Influenza Other Respir Viruses. 2023 Mar 26;17(3):e13098. doi: 10.1111/irv.13098 (PMC10040952; doi:10.1111/irv.13098)
Supplement: Supplementary file 1 — Table S1. Vaccines co‐administered with GSK's IIV4 [file IRV-17-e13098-s001.docx]

# Appendix

**Manuscript Title**: Enhanced safety surveillance of GSK's quadrivalent seasonal influenza vaccine in Germany and Spain (2021/2022 season) using an electronic patient-reported outcome system for vaccine safety remote monitoring

**Journal:** Influenza and other respiratory viruses

**Authors**: Gaël Dos Santos^1^, Tamara Eckermann^2^, Xavier Martínez‑Gómez^3^, Jose Parra^1^, Ugo Nwoji^4^, Ignacio Salamanca de la Cueva^5^

**Affiliations:**

^1^ GSK, Wavre, Belgium

^2^ Hausarztpraxis Heimeranplatz, Munich, Germany

^3^ Hospital Universitari Vall d’Hebron, Barcelona, Spain

^4^ GSK, Rockville, MD, USA

^5^ Instituto Hispalense de Pediatría, Sevilla, Spain

**Corresponding author:** Gael Dos Santos

GSK, 20 Fleming Avenue, 1300 Wavre, Belgium

Email: gael.x.dos-santos@gsk.com

Telephone: +32 1085 9141

Table S1. Vaccines co-administered with GSK’s IIV4

| **Co-administered vaccination class**† | **Germany**  **N=422** | | **Spain**  **N=555** | | **Total**  **N-977** | |
| --- | --- | --- | --- | --- | --- | --- |
|  | **n** | **%** | **n** | **%** | **n** | **%** |
| **Any** | **48** | **11.4** | **61** | **11.0** | **109** | **11.2** |
| Other viral (i.e., COVID-19) | 46 | 10.9 | 1 | 0.2 | 47 | 4.8 |
| Pneumococcal |  |  | 31 | 5.6 | 31 | 3.2 |
| Hepatitis |  |  | 25 | 4.5 | 25 | 2.6 |
| Papillomavirus |  |  | 6 | 1.1 | 6 | 0.6 |
| Meningococcal |  |  | 5 | 0.9 | 5 | 0.5 |
| Measles |  |  | 4 | 0.7 | 4 | 0.4 |
| Tetanus |  |  | 3 | 0.5 | 3 | 0.3 |
| Varicella zoster | 1 | 0.2 | 2 | 0.4 | 3 | 0.3 |
| Hemophilus influenzae b |  |  | 2 | 0.4 | 2 | 0.2 |
| Bacterial and viral, combined | 1 | 0.2 |  |  | 1 | 0.1 |
| Pertussis |  |  | 1 | 0.2 | 1 | 0.1 |

COVID-19: coronavirus disease 2019; IIV4: inactivated quadrivalent seasonal influenza vaccine; N, n: Total number, number in subcategory

†Vaccination classes coded using World Health Organization Drug Dictionary
